# Pediatric burden and seasonality of human metapneumovirus over 5 years in Managua, Nicaragua

**DOI:** 10.1111/irv.13034

**Published:** 2022-08-14

**Authors:** Kathryn Hacker, Guillermina Kuan, Nivea Vydiswaran, Gerardo Chowell‐Puente, Mayuri Patel, Nery Sanchez, Roger Lopez, Sergio Ojeda, Brenda Lopez, Jarrod Mousa, Hannah E. Maier, Angel Balmaseda, Aubree Gordon

**Affiliations:** ^1^ School of Public Health, Department of Epidemiology University of Michigan Ann Arbor Michigan USA; ^2^ Sustainable Sciences Institute Managua Nicaragua; ^3^ Centro de Salud Sócrates Flores Vivas Ministry of Health Managua Nicaragua; ^4^ School of Public Health, Department of Population Health Sciences Georgia State University Atlanta Georgia USA; ^5^ Laboratorio Nacional de Virología, Centro Nacional de Diagnóstico y Referencia Ministry of Health Managua Nicaragua; ^6^ College of Veterinary Medicine, Center for Vaccines and Immunology University of Georgia Athens Georgia USA; ^7^ College of Veterinary Medicine, Department of Infectious Diseases University of Georgia Athens Georgia USA

**Keywords:** ALRI, community‐based, human metapneumovirus, respiratory infection, seasonality

## Abstract

**Background:**

Human metapneumovirus (hMPV) is an important cause of pediatric respiratory infection. We leveraged the Nicaraguan Pediatric Influenza Cohort Study (NPICS) to assess the burden and seasonality of symptomatic hMPV infection in children.

**Methods:**

NPICS is an ongoing prospective study of children in Managua, Nicaragua. We assessed children for hMPV infection via real‐time reverse‐transcription polymerase chain reaction (RT‐PCR). We used classical additive decomposition analysis to assess the temporal trends, and generalized growth models (GGMs) were used to estimate effective reproduction numbers.

**Results:**

From 2011 to 2016, there were 564 hMPV symptomatic infections, yielding an incidence rate of 5.74 cases per 100 person‐years (95% CI 5.3, 6.2). Children experienced 3509 acute lower respiratory infections (ALRIs), of which 160 (4.6%) were associated with hMPV infection. Children under the age of one had 55% of all symptomatic hMPV infections (62/112) develop into hMPV‐associated ALRIs and were five times as likely as children over one to have an hMPV‐associated ALRI (rate ratio 5.5 95% CI 4.1, 7.4 *p* < 0.001). Additionally, symptomatic reinfection with hMPV was common. In total, 87 (15%) of all observed symptomatic infections were detected reinfections. The seasonality of symptomatic hMPV outbreaks varied considerably. From 2011 to 2016, four epidemic periods were observed, following a biennial seasonal pattern. The mean ascending phase of the epidemic periods were 7.7 weeks, with an overall mean estimated reproductive number of 1.2 (95% CI 1.1, 1.4).

**Conclusions:**

Symptomatic hMPV infection was associated with substantial burden among children in the first year of life. Timing and frequency of symptomatic hMPV incidence followed biennial patterns.

## 1 INTRODUCTION

Human metapneumovirus (hMPV) is a viral respiratory pathogen of global importance.^1^, ^2^, ^3^, ^4^ First identified in 2001, hMPV is a single‐stranded negative‐sense RNA pneumovirus which, based on serologic studies, has circulated worldwide in human populations for at least seven decades.^5^ HMPV is divided into two genetic groups: A and B, which are further differentiated into six known lineages A1, A2a, A2b, A2c, B1, and B2^6^, ^7^, ^8^ and infects all ages, with severe events occurring in children, the elderly, and the immunocompromised.^9^ In children, hMPV is pervasive in early life, causing both upper^10^ and severe lower respiratory infections.^1^, ^11^ Indeed, most children are seropositive for hMPV by age five.^5^, ^11^, ^12^, ^13^ Severe hMPV infection is also an important cause in respiratory‐associated childhood hospitalization and has globally been estimated to account for 4–18% of acute lower respiratory infection (ALRI) hospital admissions.^2^, ^14^, ^15^

Despite the importance of hMPV as a childhood respiratory infection, key questions regarding incidence, severity, and seasonality of hMPV infections particularly in lower‐ and middle‐income countries (LMIC) remain. Globally, there is significant variation in seasonality of hMPV by location, and hMPV infections can occur throughout the year.^3^, ^16^ Reinfection and repeat symptomatic episodes of hMPV in both children and adults have also been noted^12^, ^17^, ^18^, ^19^ highlighting the clinical challenge of this ubiquitous pathogen.

Currently no vaccine exists for hMPV. An important step toward this goal is describing the seasonality and burden of hMPV particularly in LMICs. While research on hMPV is increasing, there are few long‐standing cohort studies conducted in Central and Latin America.^3^ In this study, we describe the burden, symptomatic incidence rate, reinfection, and seasonality of hMPV among a cohort of children in Managua, Nicaragua. We additionally describe estimates of effective reproduction numbers for each of the epidemic peaks observed from 2011 to 2016.

## 2 METHODS

### 2.1 Study population

A detailed report on the methods and protocol used for the Nicaraguan Pediatric Influenza Cohort (NPICS) has been described previously.^20^, ^21^ Briefly, the primary aim of the NPICS study is to assess the burden, incidence, and seasonality of influenza in Nicaragua. However, while most infectious disease studies focus on a single pathogen or syndrome, NPICS was developed with the goal of assessing multiple respiratory pathogens and can test stored samples for additional pathogens. The NPICS study is an ongoing prospective cohort study initiated in 2011 and includes children aged 0–14 years, residing in District II in Managua, Nicaragua. Legal guardians are encouraged to bring their children to the Health Center Sócrates Flores Vias (HCSFV), at the first sign of illness and receive free medical care and are thus incentivized to use this health outpost as opposed to seeking care at other medical clinics. Initial enrollment for the cohort study was conducted in 2011 by randomly sampling children aged 3–11 years who were enrolled in a previous cohort study for influenza within District II, and additional children aged 0–2 were recruited through house‐to‐house visits within the catchment area. The age distribution of the NPICS cohort is representative of children of Nicaragua^20^ and spatially representative of district II in Managua.^20^ Additional children aged 0–2 years were recruited from houses throughout the study catchment area. Children ≤4 weeks old are enrolled monthly into NPICS and age out of the study on their 15th birthday. In this study, we assess those enrolled in NPICS from 2011 to 2016.

### 2.2 Case identification

Children aged 0–14 years were followed via annual surveys in addition to clinic visits where caregivers were asked to bring their children at the first sign of illness. In this study, our primary outcome is symptomatic real‐time reverse‐transcription polymerase chain reaction (RT‐PCR)‐positive hMPV cases of children brought to clinic. Samples were tested using RT‐PCR if children met specific clinical features: (1) Reported fever (37.8C) or feverishness with cough, sore throat or runny nose for children aged 2 years and older, (2) Only fever or feverishness for children under 2, (3) Severe respiratory symptoms as evaluated by a physician including wheezing, chest indrawing, and apnea, and (4) Hospitalization with respiratory symptoms or sepsis.

Our secondary outcome was hMPV‐associated acute lower respiratory infection (ALRI), which was determined as patients that presented with a diagnosis of bronchiolitis, bronchitis, bronchopneumonia, or pneumonia or bronchial hyper‐reactivity as determined by study physicians. To assess hMPV‐associated ALRI, we selected all clinic visits that met the ALRI criteria occurring up to 14 days prior to the clinic visit or 28 days after an hMPV‐positive RT‐PCR. HMPV positive tests spaced more than 30 days apart with different symptom onset dates were considered separate episodes. If the hMPV positive tests were less than 30 days apart, the first symptom onset date was used.

### 2.3 Sample collection and laboratory testing for hMPV

Nasal oropharyngeal specimens were collected for all children ≤6 months for those that met the clinical testing definitions. Combined nasal and oropharyngeal swabs were collected for children >6 months who met the testing criteria. RNA was extracted (QIAamp Viral RNA Mini Kit, Qiagen) and then tested by RT‐PCR for hMPV using CDC (Center for Disease Control) standardized protocols.^22^ Laboratory protocols were unchanged throughout the study.

### 2.4 Statistics

#### 2.4.1 Incidence calculations

Incidence rates were calculated for all symptomatic hMPV infections in addition to stratifying by age, sex, and hMPV‐associated ALRI from 2011 to 2016. Person‐time was calculated as the amount of time starting at participant enrollment to December 30, 2016, or withdrawal from the study. Withdrawal for those who were lost to follow‐up was calculated as the midpoint of the date of last contact and the date recorded by study personnel as lost to follow‐up. Incidence was calculated using generalized linear models with Poisson distributions, participant age was calculated on a weekly basis.

To assess if there were significant differences in hMPV‐associated ALRI reported by sex, we used generalized estimating equations (GEE) assuming a dependence on the individual across the sampling weeks. Weeks when hMPV‐associated ALRI was not reported was assumed to be negative for hMPV‐associated ALRI.

### 2.5 Age and time between repeat hMPV infections

To assess the association between participant age and time between repeat hMPV illnesses, we selected participants with multiple hMPV illnesses. We then calculated the interval in months between infections. We used a generalized additive model (GAM) to assess the relationship between participant age and the interval between infections. To account for right‐censoring of our data we used a cox‐proportional hazard model to estimate survival curves for those who had their first PCR detected symptomatic hMPV infection in their first 2 years of life (those who are most likely seronaive prior to symptomatic infection; Figure S2), those 3–5 years old, and those 6 or older. We used a G‐computation approach to generate adjusted survival curves for the two age‐brackets.^23^, ^24^

### 2.6 Seasonality and seasonal decomposition analysis of hMPV

We evaluated the number and incidence of weekly hMPV cases and hMPV‐associated ALRI cases from 2011 to 2016. We used R's^25^ (R Version 4.0.4) decompose (part of the stats package) and forecast package^26^ to assess the temporal dynamics of hMPV cases and specifically isolate the trend, seasonal, and error components. Because the magnitude of the seasonal fluctuations and the variation around the trend‐cycle do not vary proportionally with time, we used an additive time‐series decomposition approach to isolate the temporal trend, seasonality, and error components. In additive decomposition, we assume

$$y_{t}=S_{t}+T_{t}+R_{t}$$

where *y*_*t*_ is the data, *S*_*t*_ is the seasonal component, *T*_*t*_ is the trend component, and *R*_*t*_ is the remainder component.

### 2.7 Effective reproduction numbers

We estimated the effective reproduction number (R effective) from the initial growth phase of the local hMPV epidemics using the generalized‐growth method,^27^ which links the generation interval of the disease with the trajectory of the number of new cases per week to derive our R estimates. This method is especially useful to characterize a range of growth dynamics via two parameters: The growth rate (*r*) and the epidemic growth scaling (*p*). This growth dynamics value ranges from constant incidence (*p =* 0) to exponential growth (*p =* 1).^27^ We assumed a gamma distributed serial interval of 5 and 7.5 days and a standard deviation of 1 day.^28^, ^29^

## 3 RESULTS

### 3.1 Study participation

**FIGURE 1:**
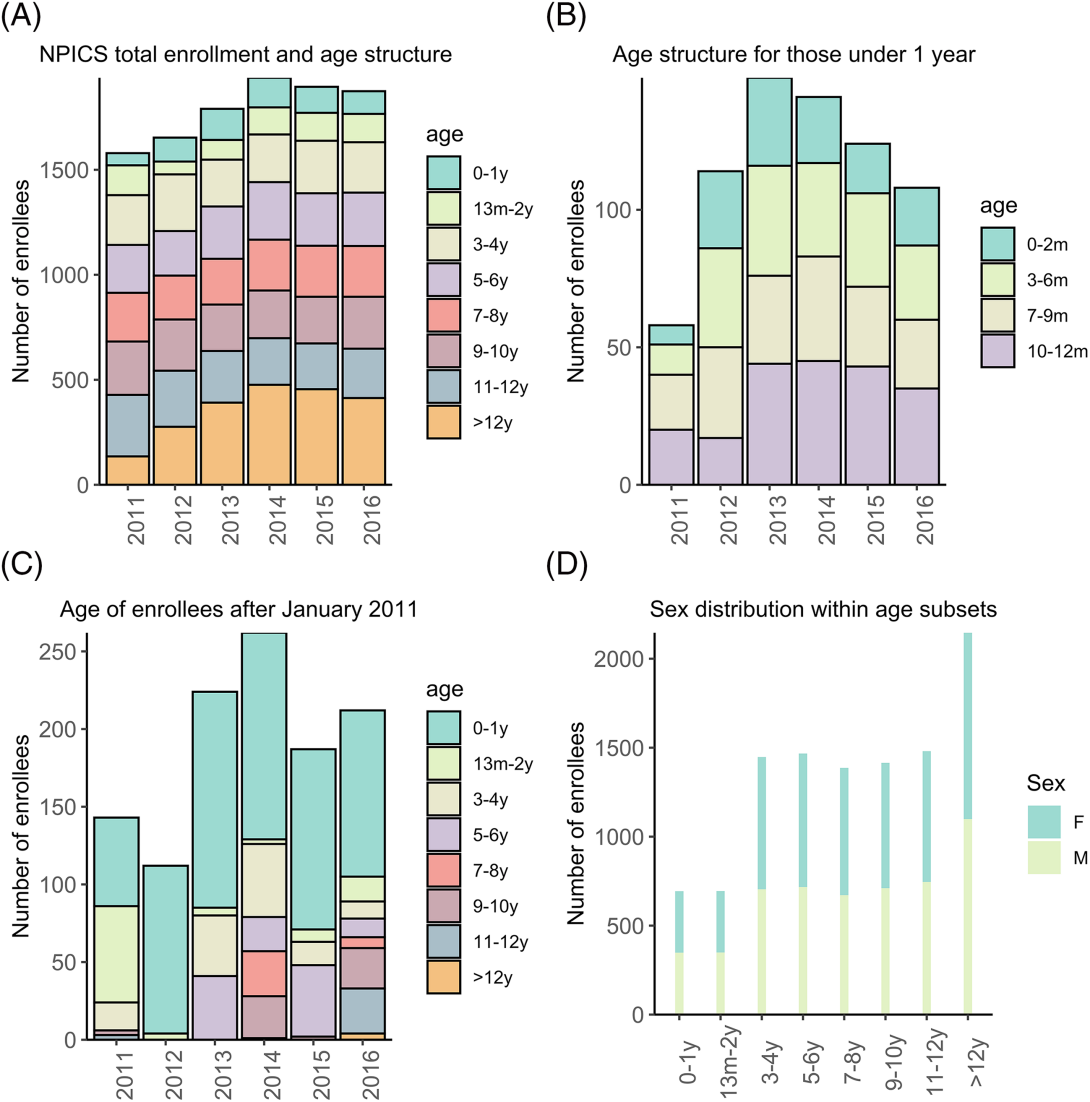
Age characteristics of the Nicaraguan Pediatric Influenza Cohort Study (NPICS) from 2011 to 2016. (A) Age of all children enrolled in NPICS from 2011 to 2016, (B) age structure of NPICS cohort for those under 1 year of age, (C) age of new enrollees by year following the initial enrollment period, (D) reported sex and age distribution of all enrollees

From 2011 to 2016, 2576 children were enrolled in NPICS, 1269 (49.3%) were boys and 1307 (49.7%) were girls. The age distribution, enrollment, and reported sex by year are summarized in Figure 1. The overall age distribution by cohort year is described in Figure 1A. The age structure for those under 1 year old are summarized in Figure 1B. The median age of those entering the cohort after January 2011 was 5 months old (IQR 0.7–29.4 months), the majority being enrolled in their first year of life (Figure 1C). The median age of enrollees exiting the cohort was 7 years (IQR 4–11 years). Study participants consistently visited the clinic throughout the study period (Figure S1), across the study period 24.8% of participant clinic visits (11 677/47006) met the hMPV testing criteria, and 9.3% (4346/47006) met the criteria for ALRI. The loss‐to‐follow up throughout the study period was low, ranging from 2%–5% per year and are described in detail in Table S1.

### 3.2 Symptomatic hMPV

**FIGURE 2:**
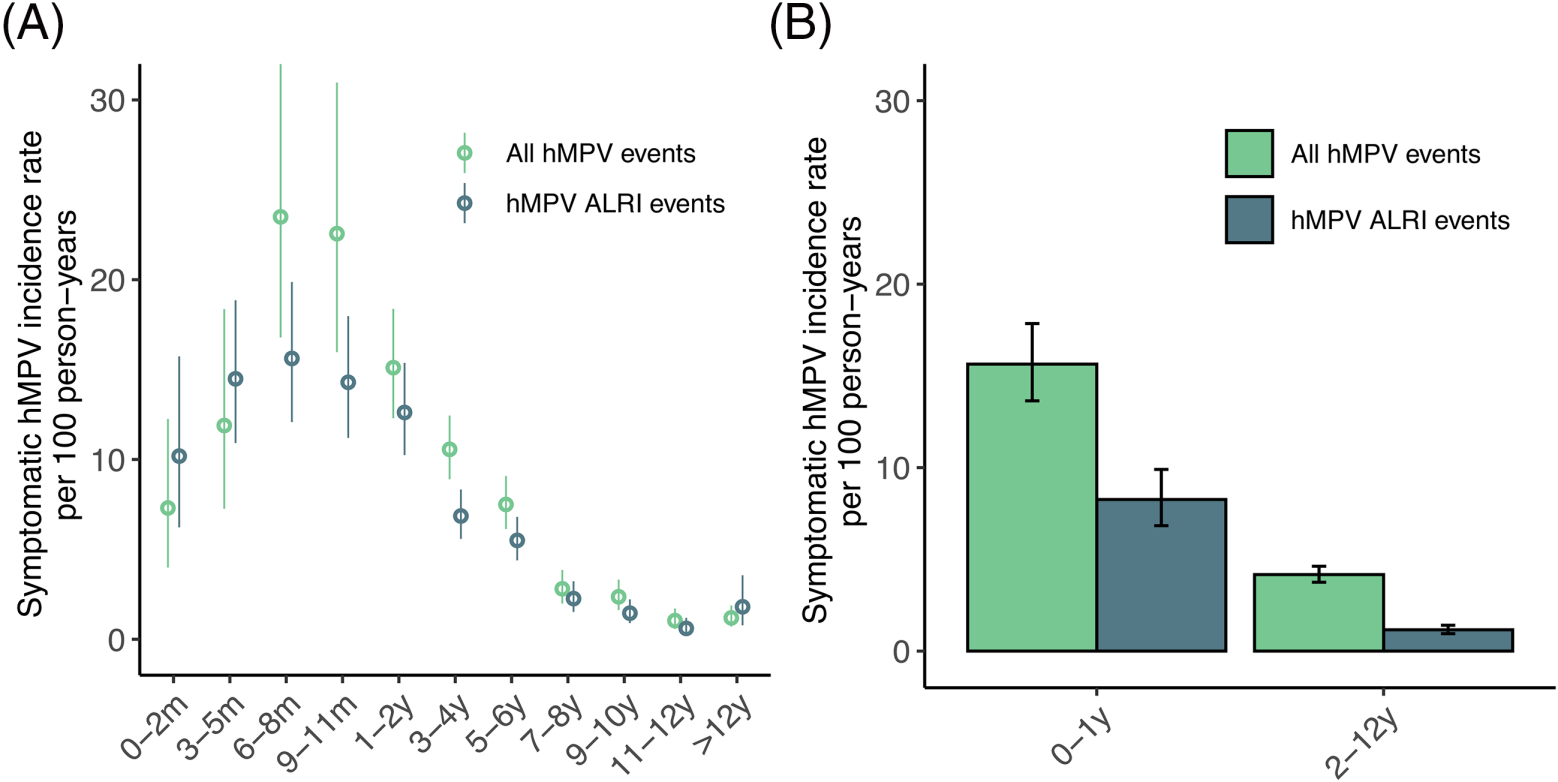
Incidence of symptomatic‐hMPV and hMPV‐associated ALRI infection by age category with 95% CI. Incidence rate per 100 person‐years of symptomatic hMPV cases and hMPV‐associated ALRIs (A) by age category and (B) stratified by 0–1 year and 2–12 years

From 2011 to 2016, of the 2576 children that participated in the cohort 478 (18.6%) had at least one RT‐PCR confirmed hMPV illness episode (Table 1). The overall symptomatic incidence rate was 5.74 (95% CI 5.3, 6.2) per 100 person‐years (Table S2). Most symptomatic hMPV illness occurred in the first year of life (Figure 2), with the highest incidence rate occurring between 6–8 months (23.5 cases per 100 person years 95% CI 16.8, 32.0). The lowest incidence was observed in the 10–12 age group (1.0 case per 100 person years 95% CI 0.6, 1.7). Children under 1 year old had 3.2 times the incidence of children 1 year or older (Rate Ratio 3.2 95% CI 2.6, 4.0 *p* < 0.001). There was no significant difference (*p* > 0.05) observed between sexes (Table S2).

**TABLE 1:** Symptomatic hMPV illness and subsequent positive symptomatic episodes. Total number of symptomatic hMPV positive infections and reinfections and summary statistics

	Number of symptomatic hMPV cases	Male sex (%)
All PCR confirmed symptomatic hMPV Cases	564	279 (49.4)
Primary symptomatic episode	478	240 (50.0)
Secondary symptomatic episode	79	34 (44.2)
Tertiary symptomatic episode	7	3 (42.9)
Quaternary symptomatic episode	1	1 (100)

**FIGURE 3:**
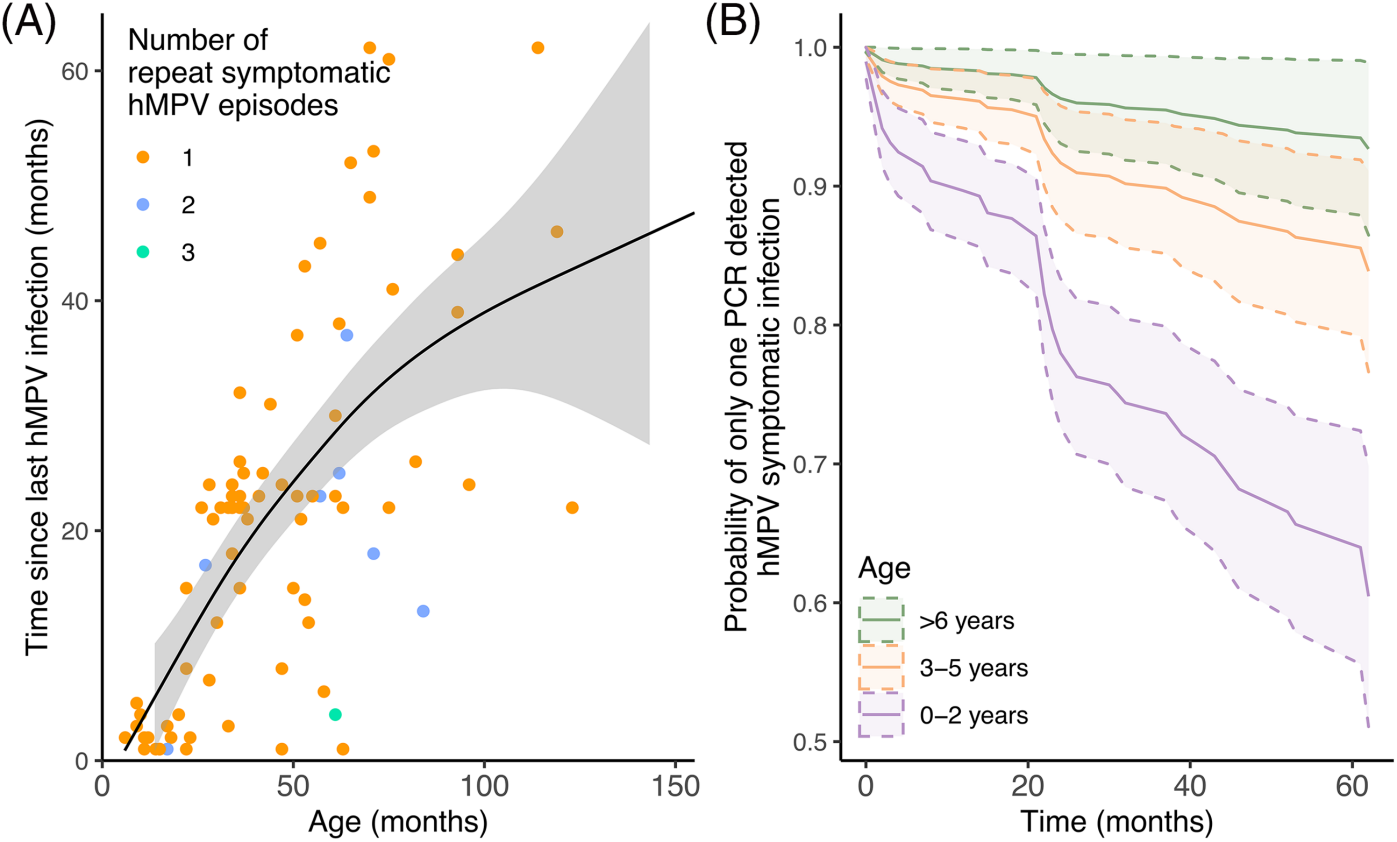
Age of patients with PCR detected repeat symptomatic‐hMPV infections compared with the time since previous infection. (A) Fitted generalized additive model (GAM) comparing age of illness in months and the amount of time since previous infections not accounting for right censoring. (B) Adjusted survival curve using direct adjustment to assess the probability of only having one PCR‐detected symptomatic hMPV reinfection given the age group at first symptomatic infection accounting for right censoring. Age groups are stratified by 0–2 years of age (most likely to be seronaïve prior to first PCR‐detected symptomatic hMPV infection based on serology Figure S2), 3–5 years of age, and those over 6.

Of the total 564 hMPV symptomatic cases, 87 (15%) were detected symptomatic reinfections (Table 1, Figure 3). To determine an age cut‐point for children likely to be experiencing a first infection we examined hMPV antibodies in a subset of the cohort, which is detailed in Supplemental Methods and Figure S2. However, in a subset 34 children who had a symptomatic hMPV event recorded in the cohort (Supplemental Methods, Figure S2), only 20% of children in this subset ever presented a seronegative, and all were 2 years old or younger. Thus, children under 2 at first symptomatic infection, were more likely experiencing a first infection, while those 3 and older were likely to be experiencing a second or subsequent infection. Without accounting for right‐sided censoring, the median time to detected symptomatic reinfection was 22 months, or 1.8 years (min 1 month, max 5.1 years), and the median age of detected symptomatic reinfection was 40 months, or 3.3 years (min 6 months, max 10.2 years). Participant age was significantly associated with the amount of time between positive RT‐PCRs (*p* < 0.001) (Figure 3A). Children 2 years old or younger at first detected symptomatic infection (the only population likely to be seronaive in the study cohort; Figure S2) had the greatest probability to experience a symptomatic reinfection (Figure 3B). Indeed, the probability of secondary symptomatic infection was significantly higher for those whose first infection occurred in their first 2 years of life compared with symptomatic infections occurring later in life (Figure 3B).

### 3.3 hMPV‐associated ALRI

During the study period, 3509 ALRI events were recorded at the study clinics. Of those events, 160 (4.6%) were associated with hMPV. Out of the total 564 hMPV positive cases, 160 (28%) were associated with an ALRI diagnosis (Table S2). The incidence rate for hMPV‐associated ALRI was 2.1 cases per 100 person‐years (95% CI 1.9, 2.4). Of the 160 ALRI events, 7 (4.4%) were severe enough to transfer the children to a hospital for further treatment. Like symptomatic hMPV cases, there were more ALRI events reported in males (*N* = 116, 42% of males with hMPV) in our study compared with females (*N* = 94, 33% of females with hMPV), however this difference was not significant. There was a significant difference in incidence rate observed between hMPV‐associated ALRI episodes in those under 1 year of age compared with those over a year (rate ratio 5.5 95% CI 4.1, 7.4 *p* < 0.001). For children under the age of one, 55% of all symptomatic hMPV episodes resulted in an ALRI event compared with just 33% for children over one.

### 3.4 Seasonality and seasonal decomposition analysis of hMPV

**FIGURE 4:**
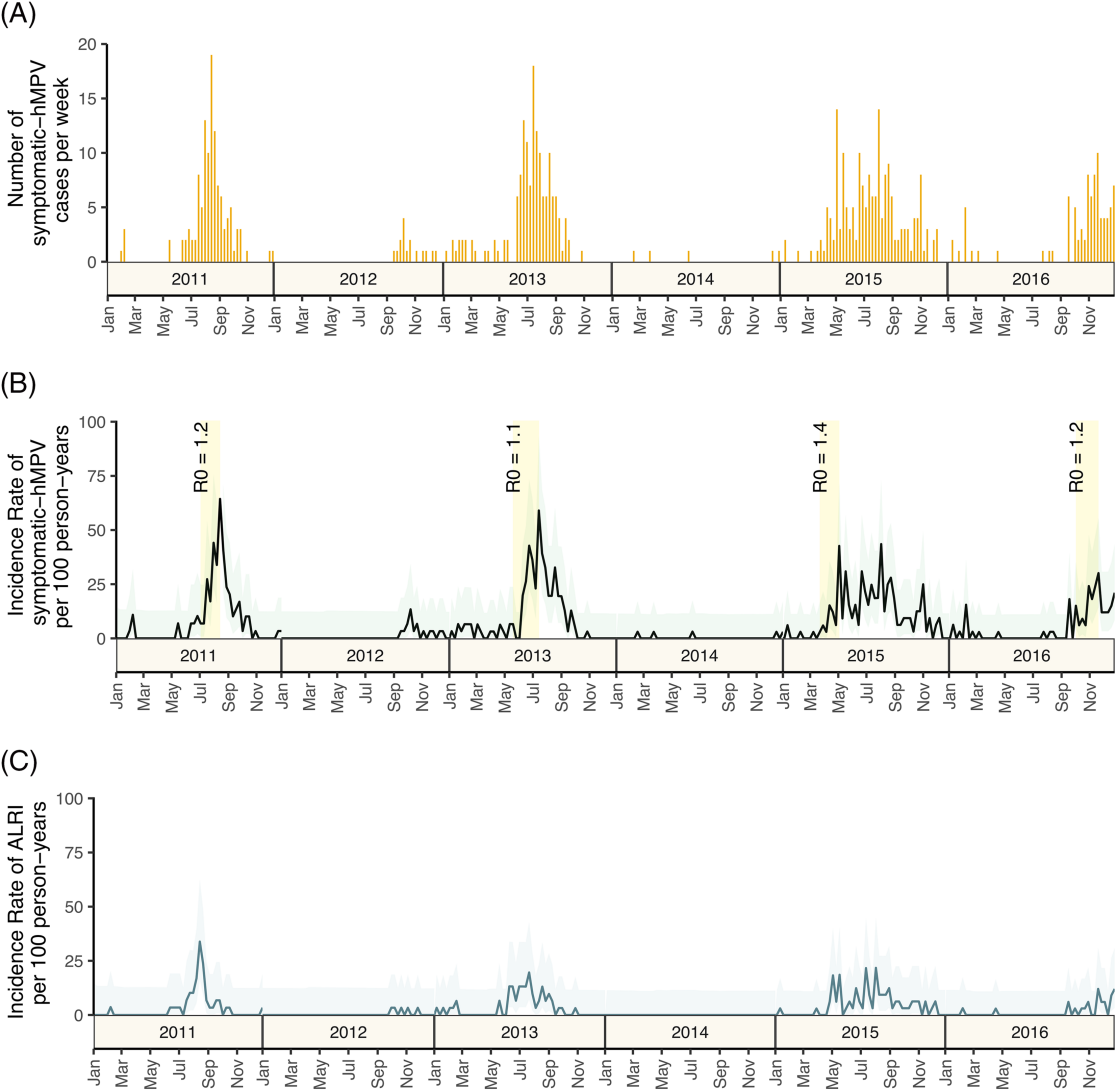
Weekly hMPV case counts and symptomatic incidence rate from 2011 to 2016. (A) Weekly count of hMPV cases in the NPICS cohort from 2011 to 2016. (B) Weekly symptomatic incidence rate for hMPV cases in the NPICS cohort within a 95% confidence interval from 2011 to 2016. Yellow bars indicate the ascending phase length for each outbreak and the subsequent estimated mean effective reproductive number based on a 5‐day serial number. (C) Weekly symptomatic incidence of hMPV cases causing acute lower‐respiratory infection (ALRI) in the NPICS cohort within a 95% confidence interval from 2011 to 2016

HMPV epidemics occurred in alternate years (Figure 4). Years with the highest hMPV incidence were 2011, 2013, and 2015. Cases tended to peak during July–August, however there was additional variation of incidence throughout the year, which was particularly notable in 2015 (Figure 4 and Figure S3). In 2012 and 2014 there was no major epidemic. There was a rise in case numbers in 2016 in September–January which differs from peaks in previous years, however it is possible that the peak epidemic period occurred later into 2017 (Figure 4, Figure S3).

**FIGURE 5:**
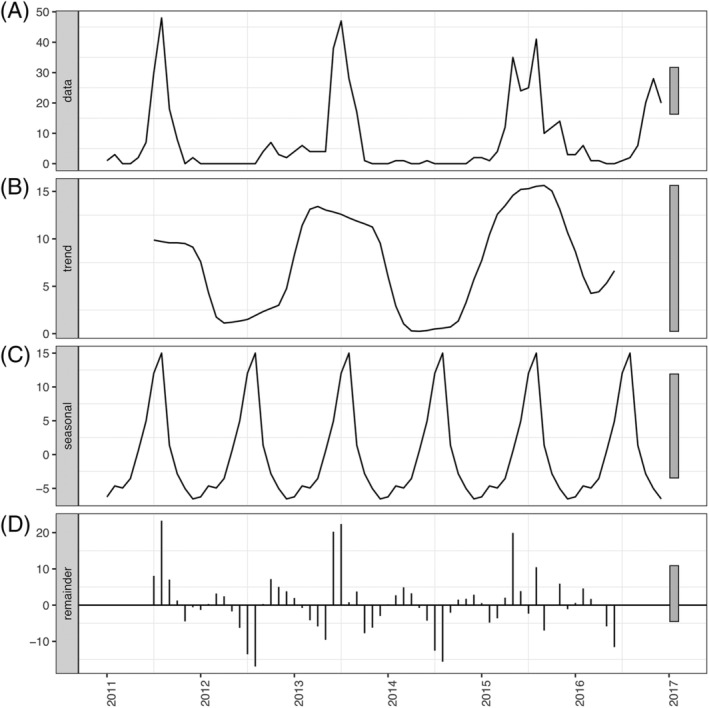
Classical additive decomposition of monthly hMPV infections. Panel A shows the original non‐detrended data of symptomatic hMPV episodes per month from 2011 to 2017. Panel B shows the trend‐cycle component for monthly data (seasonal and remainder components extracted). Panel C shows the seasonal component extracted from the original data. Panel D shows the remainder component when the trend‐cycle and seasonal component are extracted. The grey bars to the right of the panels denote the scales of each of the components.

When the trend (*T*_*t*_) was isolated from the seasonal and remainder components in classical additive decomposition, there was no overall observable increase or decrease in the number of monthly symptomatic hMPV cases (Figure 5B). When the seasonality and remainder were extracted from the trend there was a distinct decrease of the number of cases in 2012 and 2014 (Figure 5B), and an increase in cases in 2013 and 2015. This trend was also noted in the remainder component where there were significant increases and decreases that were not explained by the trend (*T*_*t*_) or seasonal component (*S*_*t*_) (Figure 5C,D). The increases in symptomatic hMPV cases when annual seasonal patterns (*S*_*t*_) and longitudinal trend were extracted (*T*_*t*_) revealed a biennial residual pattern.

### 3.5 Effective reproduction numbers

Four main epidemic periods were observed (in 2011, 2013, 2015, and 2016) and are described in detail in Figure 4 and Table S3. The mean ascending phase of the epidemic periods were 7.7 weeks. Effective reproduction numbers ranged from 1.1–1.7 with a mean effective reproduction number of 1.3, depending on epidemic period and estimated serial interval (Figure 4B, Table S3).

## 4 DISCUSSION

In this prospective cohort, we demonstrate substantial burden of symptomatic‐hMPV infection in children (5.7 cases per 100 person‐years) and is an important cause of ALRI particularly for children in their first year of life. While the seroprevalence of hMPV in children varies globally, ranging from <5% to >30%,^3^ the overall incidence among all children in the Nicaraguan cohort is one of the highest recorded in Central and South America.^30^, ^31^, ^32^, ^33^, ^34^, ^35^ Few prospective cohort studies assess for hMPV in children, and studies that screen for hMPV primarily occur at surveillance hospitals making precise comparisons of incidence across communities and countries challenging. To our knowledge, this study is the longest running clinical‐based community cohort in Central or South America assessing hMPV in children.

In the Nicaraguan study cohort, the majority of symptomatic‐hMPV and critically hMPV‐associated ALRI occurred in first year of life (Figure 3). Those under 1 year old were 3.2 times more likely to have a symptomatic hMPV episode compared with those aged 1–14. Worldwide hMPV infection is greatest in those <5 years old,^2^, ^3^ however there is considerable variation in age and infection in children under five.^11^, ^36^, ^37^, ^38^ However, in long‐running prospective cohort studies in children the burden appears to primarily affect those under a year of age.^36^, ^39^ Recent global modeling studies indicate that infants under 1 year have disproportionally high risks for hMPV‐associated ALRI similar to Respiratory Syncytial Virus (RSV) and influenza.^2^ Indeed, the highest incidence for influenza‐associated ALRI for children aged 9–11, in the same study cohort was 4.8 influenza‐associated ALRI cases (95%CI: 2.8–8.3) per 100 person‐year compared with 13.7 hMPV‐associated ALRI per 100 person‐year for children of the same age (95% CI: 8.7–20.6).^20^ This study also indicates that children under a year 6 months old in LMICs are at an increased risk of death compared with upper‐middle‐income countries. In the United States, a long‐running cohort found that the hMPV infection was greatest in those under 1 year of age.^39^ Similarly, in Guatemala, a hospital‐based cohort similar in size and scope to our study, also found increasing hMPV incidence throughout the first year of life.^36^ Our study demonstrates that not only are children at a high risk of acquiring symptomatic hMPV, but also symptomatic infection with hMPV is likely to result in an ALRI event, particularly for infants under a year old.

We additionally found that symptomatic reinfection of hMPV was common. While this study did not capture asymptomatic reinfections, the total number of symptomatic reinfections is substantial. Indeed, based on the subset of children whose serology was evaluated, only those two or younger were seronaive. It is therefore likely that symptomatic infections captured in this study by children older than two are likely experiencing reinfections. Globally reinfection is common, likely due to poor development of T and B cell immunological memory or a lack of sterilizing immunity.^12^, ^17^, ^18^, ^19^ Additionally, changes in the predominate circulating strain or co‐circulating strains or viral evolution may also have implications for sterilizing immunity, reinfection dynamics, and age at symptomatic reinfection, but without additional genetic information and asymptomatic infection it is challenging to assess this question directly. However, we demonstrate that reinfection of symptomatic hMPV is common, and more likely to occur in younger age groups. This pattern of symptomatic infections occurring early in life and decreasing with age may demonstrate growing immunity as children become exposed to various strains in early life. These early exposures may only confer partial immunity, however as children become reinfected they continue to build immunity which may result in fewer symptomatic infections as they age, a pattern found in other viral infections.^40^, ^41^, ^42^

Similar to other studies, hMPV‐associated ALRI events accounted for a substantial proportion of symptomatic hMPV episodes.^3^, ^11^, ^39^ Throughout the study period, hMPV‐associated ALRI constituted 27% ‐ 43% of all symptomatic hMPV infections. The likelihood of hMPV‐associated ALRI was five‐times higher in children under the age of one compared with those older than one. This severity is consistent with other hospital cohort studies.^15^ In the United States, the annual rate of hospitalization was highest for infants in the 0‐ to 5‐month range.^15^

Seasonality of symptomatic hMPV varied considerably year to year. While hMPV infection occurred throughout the study period, four epidemic peaks were identified. Effective reproductive numbers varied based on year and depending on the estimate of serial interval used. We were unable to find other published estimates of the reproductive number for hMPV and while better estimates of generation interval are needed for more precise estimates, this study is an important step forward in estimating the potential spread of pediatric hMPV. During the epidemic periods observed, cases peaked in July or August corresponding to the rainy season which lasts from June to November. This seasonality is similar to other studies conducted in tropical and subtropical areas where epidemic peaks tended to occur during periods of high rainfall and high relative humidity,^37^, ^43^, ^44^ in contrast to temperate areas where hMPV infection predominately peaks in the winter and spring months.^3^ Globally, seasonality of hMPV is broadly influenced by climatic features, but local metrological conditions likely influence variation regionally and locally.^3^, ^16^

While longer time scales are needed to assess fixed patterns in seasonality, biennial seasonality occurred during the first 4 years. While most studies observe annual hMPV epidemic cycles,^16^, ^31^, ^36^, ^37^, ^38^, ^44^, ^45^, ^46^, ^47^, ^48^ biennial seasonality in hMPV infection is uncommon,^49^ and has not been observed in the tropics. For some infectious diseases, like measles, periodicity resulting in biennial transmission is due to the variation of the proportion of susceptible individuals in a population.^50^, ^51^ While age structure and distribution of those entering the cohort was stable throughout the study period, we are unable to broadly assess if the total number of susceptible individuals are changing and if this change influences the seasonality of hMPV.

This study was not without limitations. While this study is longer compared with many cohort studies on hMPV, it is not long enough to describe temporal patterns accurate of seasonal dynamics. Additionally, we did not assess genetic variation in hMPV, which might offer insight into seasonal dynamics, disease severity, and reinfection dynamics. Based on current literature, there are no strong associations between these lineages and disease severity, or when detected, were found in smaller studies lacking substantial power.^16^, ^45^, ^48^, ^52^ It is likely that multiple lineages and changes in lineage are occurring during the study period. Studies assessing the seasonality of subgroup types have found alternating subgroup seasonality, with subgroup dominance shifting every 1–3 years while the clinical presentation of hMPV remained unchanged. This is consistent with our study where hMPV‐associated ALRI events were consistently proportional to the number of symptomatic events. It is therefore unlikely that changes in lineage effect the number of severe ALRI outcomes. While specific genetic groupings are globally more common in specific regions, for example in Asian countries after 2005 A2c and A2b genetic groupings were more common,^6^ than samples derived from Europe, multiple lineages circulating in a specific season are common and found globally^3^, ^6^, ^10^, ^15^, ^16^, ^30^, ^31^, ^38^, ^45^, ^47^, ^53^, ^54^, ^55^, ^56^, ^57^ without substantial changes to their yearly seasonal dynamics.

hMPV is a ubiquitous childhood respiratory illness. While seroprevalence for hMPV is high, globally little is known about hMPV in Latin America or how its dynamics might influence prevention, prediction, and surveillance of hMPV. Here, we demonstrate that hMPV infection is an important cause of ALRI in children and is particularly important for children under 1 year of age. While hMPV infections occur throughout the year, distinct biennial seasonality for hMPV infection was evident in our cohort and may be important in defining timing of future interventions.

## CONFLICT OF INTEREST

Aubree Gordon serves on an advisory board for Janssen. No conflict of interest declared by other authors.

## AUTHOR CONTRIBUTIONS

**Kathryn Hacker:** Conceptualization; formal analysis; methodology; validation; visualization. **Guillermina Kuan:** Data curation; project administration; resources. **Nivea Vydiswaran:** Data curation; project administration. **Gerardo Chowell‐Puente:** Formal analysis; methodology; validation; visualization. **Mayuri Patel:** Data curation; project administration. **Nery Sanchez:** Data curation; project administration. **Roger Lopez:** Data curation; project administration. **Sergio Ojeda:** Data curation; project administration. **Brenda Lopez:** Data curation; project administration. **Jarrod Mousa:** Conceptualization; data curation; funding acquisition; methodology; project administration; resources. **Hannah Maier:** Formal analysis; methodology; validation; visualization. **Angel Balmaseda:** Data curation; project administration; resources. **Aubree Gordon:** Conceptualization; funding acquisition; methodology; project administration; resources; supervision; validation.

## ETHICS STATEMENT

The study was approved by the institutional review boards at the Nicaraguan Ministry of Health, the University of Michigan, and the University of California, Berkeley.

## PATIENT CONSENT STATEMENT

Parent or guardian consent was obtained for all participants. Additionally, verbal assent was obtained for participants aged ≥6 years. All consenting documents and scripts was approved by the institutional review boards at the Nicaraguan Ministry of Health, the University of Michigan, and the University of California, Berkeley.

## PERMISSION TO REPRODUCE MATERIAL FROM OTHER SOURCES

This manuscript does not contain copyrighted works

### PEER REVIEW

The peer review history for this article is available at https://publons.com/publon/10.1111/irv.13034.

## Supporting information


**Figure S1.** Study participant clinic visits and outcomes. Panel A describes the total monthly counts of clinic visits (grey), visits that met the hMPV testing criteria (purple), and visits with ALRI (light red). Panel B describes hMPV‐related outcomes per month where visits that met the hMPV testing criteria (grey), followed by those that were symptomatic RT‐PCR confirmed hMPV events (orange), and visits with hMPV‐associated ALRI (light blue).

**Figure S2.** Serology of 34 individuals within the study cohort who had a detected PCR‐confirmed symptomatic hMPV infection during the study period. NC denotes a negative control (CA09–40) which was used as baseline signal for Area Under the Curve (AUC) values. PC denotes the positive control (anti‐hMPV F monoclonal antibody MPE8). The panel colors indicate when the symptomatic infection was detected by the study cohort team. All available annual timepoints for each of the 34 children were pulled to assess serostatus across the course of the cohort study. While all efforts were made to obtain an annual blood draw for each child, some children were missing years, or the sample was not sufficient for serology analysis. All available years for each of the 34 children are represented in the figure.

**Figure S3.** Time series patterns of monthly symptomatic hMPV infections. Panel A describes the variation of hMPV infection over time with each year plotted separately. Panel B aggregates all the years of data and plots the time series recorded by month; the blue line denotes the mean of the hMPV infections per month.

**Table S1.** Participants entering and exiting in the study cohort by year and reason for exiting the cohort.
**Table S2**. Incidence rate of symptomatic‐hMPV and hMPV‐associated ALRI in the NPICS cohort from 2011–2016 by year and reported sex.
**Table S3.** Estimated effective reproduction number for each outbreak period. We assumed a sigma of 1 day and calculated the Growth rate (*r*), scaling growth (*p*), and effective reproduction number for each outbreak using a 5‐day and 7.5‐day generation interval.

## Data Availability

Individual‐level data may be shared with outside investigators following University of Michigan IRB approval. Please contact Aubree Gordon (gordonal@umich.edu) to arrange for data access.
