# Supplementary material for: Pediatric burden and seasonality of human metapneumovirus over 5 years in Managua, Nicaragua
Source: Influenza Other Respir Viruses. 2022 Aug 14;16(6):1112–21. doi: 10.1111/irv.13034 (PMC9530515; doi:10.1111/irv.13034)
Supplement: Supplementary file 4 — Table S1. Participants entering and exiting in the study cohort by year and reason for exiting the cohort. Table S2. Incidence rate of symptomatic‐hMPV and hMPV‐associated ALRI in the NPICS cohort from 2011–2016 by year and reported sex. Table S3. Estimated effective reproduction number for each outbreak period. We assumed a sigma of 1 day and calculated the Growth rate (r), scaling growth (p), and effective reproduction number for each outbreak using a 5‐day and 7.5‐day generation interval. [file IRV-16-1112-s004.docx]

Supporting methods

Serology Data

A subset of 34 children with a known PCR-detected symptomatic hMPV infection were selected for serology. All annual blood draws that were available for the selected children were used for the analysis. To assess the serostatus of each child per timepoint, 384-well plates (Greiner) were coated with monomeric cleaved recombinant hMPV B2 F protein generated as previously described. Plates were washed once with water, then blocked with 2% blocking buffer (PBS + 2% non-fat dry milk (Bio-Rad) + 2% goat serum + 0.05% Tween-20) for 1hr at room temperature. Serum or plasma samples were diluted 1/100 in blocking and serially

diluted 2-fold. Plates were washed three times with water, and 25 μL of serum samples were

added to each well. Plates were incubated at room temperature for 1 hr, and then washed

three times with water. Goat anti-human IgG Fc-AP secondary antibody (Southern Biotech),

diluted 1:4000 in 1% blocking buffer (1:1 dilution of PBS and 2% blocking buffer), was added

and plates were incubated at room temperature for 1 hr. Plates were then washed five times

with PBS-T (PBS + 0.05% Tween-20). p-Nitrophenyl phosphate (PNPP) substrate, diluted in

substrate buffer (1.0 M Tris + 0.5 mM MgCl2, pH=9.8) to 1 mg/mL, was added, and plates were

incubated for 1 hr and read at 405 nm on a BioTek plate reader. Area under the curve values

were calculated in Graphpad Prism using the signal from an anti-influenza monoclonal

antibody, CA09-40, diluted to 20 μg/mL and assessed on each plate, as the baseline signal. The

anti-hMPV F monoclonal antibody MPE8 was used as a positive binding control.

Supplemental Tables

**Supplemental Table 1.** Participants entering and exiting in the study cohort by year and reason for exiting the cohort.

| Year | Enrolled | Withdrawn | Lost to Follow Up | Died | Total Entering | Total Exiting |
| --- | --- | --- | --- | --- | --- | --- |
| 2011 | 1578 | 3 (0.19%) | 33 (2.1%) | 1 | 143† | 37 |
| 2012 | 1653 | 14 (0.85%) | 73 (4.4%) | 1 | 113 | 88 |
| 2013 | 1790 | 13 (0.72%) | 102 (5.7%) | 2 | 225 | 117 |
| 2014 | 1938 | 32 (1.6%) | 90 (4.6%) | 1 | 265 | 233 |
| 2015 | 1894 | 22 (1.2%) | 90 (4.8%) | 0 | 188 | 233 |
| 2016 | 1874 | 18 (0.95%) | 36 (1.9%) | 1 | 213 | 149 |

†Total enrolled after the initial January start date

**Supplemental Table 2**. Incidence rate of symptomatic-hMPV and hMPV-associated ALRI in the NPICS cohort from 2011–2016 by year and reported sex.

|  |  | Symptomatic hMPV-episodes | | hMPV- associated ALRI episodes | |
| --- | --- | --- | --- | --- | --- |
|  | Person years | Cases | Incidence  (95% CI per 100 person-years) | Cases | Incidence  (95% CI per 100 person-years) |
| All | 9797.9 | 564 | 5.74 (5.3, 6.2) | 160 | 2.1 (1.9, 2.4) |
| Year |  |  |  |  |  |
| 2011 | 1506.5 | 119 | 7.9 (6.6, 9.5) | 46 | 3.1 (2.3, 4.1) |
| 2012 | 1568.3 | 16 | 1.0 (0.6, 1.7) | 5 | 0.3 (0.1, 0.8) |
| 2013 | 1613.2 | 153 | 9.5 (8.1, 11.1) | 56 | 3.5 (2.7, 4.5) |
| 2014 | 1697.8 | 5 | 0.3 (0.1, 0.7) | 0 | 0 (NA) |
| 2015 | 1689.4 | 183 | 10.8 (9.4, 12.5) | 79 | 4.7 (3.8, 5.8) |
| 2016 | 1722.7 | 88 | 5.1 (4.1, 6.3) | 24 | 1.4 (0.9, 2.1) |
| Sex |  |  |  |  |  |
| Male | 4875.8 | 279 | 5.7 (5.1, 6.4) | 116 | 2.4 (2.0, 2.4) |
| Female | 4922.1 | 285 | 5.8 (5.1, 6.5) | 94 | 1.9 (1.6, 2.3) |
| Age |  |  |  |  |  |
| 0-2 months | 191.1 | 14 | 7.3 (4.0, 12.3) | 8 | 4.2 (1.8, 8.2) |
| 3–5 months | 167.7 | 20 | 11.9 (7.3, 18.4) | 9 | 5.4 (2.5, 10.2) |
| 6–8 months | 169.8 | 40 | 23.6 (16.8, 32.1) | 22 | 13.0 (8.1, 19.6) |
| 9–11 months | 167.8 | 38 | 22.7 (16.0, 31.1) | 23 | 13.7 (8.7, 20.6) |
| 1–2 years | 659.4 | 100 | 15.2 (12.3, 18.4) | 50 | 7.6 (5.6, 10.0) |
| 2–4 years | 1349.6 | 143 | 10.6 (8.9, 12.5) | 51 | 3.8 (2.8, 5.0) |
| 4–6 years | 1395.8 | 105 | 7.5 (6.2, 9.1) | 30 | 2.1 (1.5, 3.1) |
| 6–8 years | 1349.9 | 38 | 2.8 (2.0, 3.9) | 12 | 0.9 (0.5, 1.6) |
| 8–10 years | 1392.3 | 33 | 2.4 (1.6, 3.3) | 3 | 0.2 (0.0, 0.6) |
| 10–12 years | 1445.6 | 15 | 1.0 (0.6, 1.7) | 1 | 0.1 (0.0, 0.4) |
| >12 years | 1509.0 | 18 | 1.2 (0.7, 1.9) | 1 | 0.1 (0.0, 0.4) |

**Supplemental Table 3.** Estimated effective reproduction number for each outbreak period. We assumed a sigma of 1 day and calculated the Growth rate (*r*), scaling growth (*p*), and effective reproduction number for each outbreak using a 5-day and 7.5-day generation interval.

| 5-Day Mean Generation Interval | | | | |
| --- | --- | --- | --- | --- |
| Week | Ascending phase length (weeks) | Growth rate (*r*)  Mean (95% CI) | Scaling of growth (*p*)  Mean (95% CI) | Reproduction number  Mean (95% CI) |
| 7/3/2011 | 7 | 1.50 (0.56, 3.00) | 0.66 (0.38, 1.00) | 1.20 (1.10, 1.50) |
| 5/19/2013 | 9 | 1.20 (0.48, 2.40) | 0.65 (0.40, 0.95) | 1.10 (1.10, 1.30) |
| 3/22/2015 | 7 | 0.65 (0.51, 1.10) | 0.93 (0.64, 1.00) | 1.40 (1.10, 1.50) |
| 10/2/16 | 8 | 0.74 (0.28, 2.40) | 0.76 (0.31, 1.00) | 1.20 (1.00, 1.30) |
| 7.5-Day Mean Generation Interval | | | | |
| Week | Ascending phase length (weeks) | Growth rate (*r*)  Mean (95% CI) | Scaling of growth (*p*)  Mean (95% CI) | Reproduction number  Mean (95% CI) |
| 7/3/2011 | 7 | 1.50 (0.56, 3.00) | 0.66 (0.38, 1.00) | 1.30 (1.10, 1.80) |
| 5/19/2013 | 9 | 1.20 (0.48, 2.40) | 0.65 (0.40, 0.95) | 1.20 (1.10, 1.40) |
| 3/22/2015 | 7 | 0.65 (0.51, 1.10) | 0.93 (0.64, 1.00) | 1.70 (1.30, 1.90) |
| 10/2/16 | 8 | 0.74 (0.28, 2.40) | 0.76 (0.31, 1.00) | 1.30 (1.10, 1.40) |
|  |  |  |  |  |

Supplemental Figures

**Supplemental Fig 1.** Study participant clinic visits and outcomes. Panel A describes the total monthly counts of clinic visits (grey), visits that met the hMPV testing criteria (purple), and visits with ALRI (light red). Panel B describes hMPV-related outcomes per month where visits that met the hMPV testing criteria (grey), followed by those that were symptomatic RT-PCR confirmed hMPV events (orange), and visits with hMPV-associated ALRI (light blue).

**Supplemental Fig 2.** Serology of 34 individuals within the study cohort who had a detected PCR-confirmed symptomatic hMPV infection during the study period. NC denotes a negative control (CA09-40) which was used as baseline signal for Area Under the Curve (AUC) values. PC denotes the positive control (anti-hMPV F monoclonal antibody MPE8). The panel colors indicate when the symptomatic infection was detected by the study cohort team. All available annual timepoints for each of the 34 children were pulled to assess serostatus across the course of the cohort study. While all efforts were made to obtain an annual blood draw for each child, some children were missing years, or the sample was not sufficient for serology analysis. All available years for each of the 34 children are represented in the figure.

S**upplemental Fig 3.** Time series patterns of monthly symptomatic hMPV infections. Panel A describes the variation of hMPV infection over time with each year plotted separately. Panel B aggregates all the years of data and plots the time series recorded by month; the blue line denotes the mean of the hMPV infections per month.
